# Unraveling the genetic collagen connection: clinical and therapeutic insights on genetic connective tissue disorders

**DOI:** 10.1186/s42358-024-00373-z

**Published:** 2024-04-25

**Authors:** Nilton Salles Rosa Neto, Ivânio Alves Pereira, Flávio Roberto Sztajnbok, Valderílio Feijó Azevedo

**Affiliations:** 1 Centro de Doenças Raras e da Imunidade, Hospital Nove de Julho, Rua Peixoto Gomide, 285, Cerqueira César, São Paulo, CEP 01409-001 SP Brazil; 2 Universidade Santo Amaro, São Paulo, Brazil; 3 Universidade do Sul de Santa Catarina, Florianópolis, Brazil; 4 Department of Pediatrics, Universidade Federal do Rio de Janeiro, Rio de Janeiro, Brazil; 5 Department of Internal Medicine, Universidade Federal of Paraná, Curitiba, Brazil

**Keywords:** Collagen, Extracellular matrix, Connective tissue disease, Osteogenesis imperfecta, Ehlers–Danlos syndrome, Marfan syndrome, Loeys–Dietz syndrome, Stickler syndrome

## Abstract

Hereditary connective tissue disorders include more than 200 conditions affecting different organs and tissues, compromising the biological role of the extracellular matrix through interference in the synthesis, development, or secretion of collagen and/or its associated proteins. The clinical phenotype includes multiple signs and symptoms, usually nonspecific but of interest to rheumatologists because of musculoskeletal involvement. The patient´s journey to diagnosis is long, and physicians should include these disorders in their differential diagnoses of diseases with systemic involvement. In this review, insights for the diagnosis and treatment of osteogenesis imperfecta, hypermobility spectrum disorder/Ehlers–Danlos syndrome, Marfan, Loeys–Dietz, and Stickler syndromes are presented.

## Introduction

Hereditary connective tissue disorders (HCTD) may arise from defects in the biosynthesis, assembly, posttranslational modification, secretion, or other processes involved in normal collagen formation, including enzymes and accessory proteins (for example, fibrillin, for which pathogenic variants cause Marfan syndrome – MFS) [1–3]. 

There are more than 200 conditions that may be characterized as HCTD. These disorders comprise a complex group of generally low-prevalence diseases that manifest variable clinical signs and symptoms and can be encountered in different specialties, such as rheumatology, orthopedics, nephrology, ophthalmology, dermatology, angiology, and gastroenterology. Furthermore, genetic characteristics such as pleiotropism and variable expressivity, in addition to epigenetic and environmental factors, contribute to increasing not only the diagnostic but also the therapeutic challenge [2, 4]. 

Often, these conditions involve overlapping symptoms and treatments, and physicians should be able to recognize patients with personal and/or family history of one or more of these conditions: joint or spine deformity (e.g. scoliosis, spondylolisthesis), joint hypermobility or deformity (e.g. plain pes planus), unusually short or tall stature, skin hyperextensibility, tissue fragility, poor wound healing, easy bruising, arterial dilatation or dissection, intestinal or uterine spontaneous rupture, unexplained organ infarction, multiple fractures from minor or no trauma, lens dislocation, abnormality of the cornea, spontaneous pneumothorax, or hearing loss [2]. 

## The extracellular matrix

The extracellular matrix is a dynamic structure composed of a mixture of water and collagens, proteoglycans, elastic proteins, and noncollagenous glycoproteins. The composition of organs and tissues differs, and organs and tissues are adjusted to meet the biological requirements of each organ (flexibility, resistance, structural framework).

Collagens are protein molecules composed of amino acids that follow a predefined sequence and are characterized by the formation of trimers (three chains), which are later organized into fibers. They are abundant in organisms, particularly in the extracellular matrix, and are also essential for homeostasis, morphogenesis, and tissue differentiation [3, 5–7]. 

The collagen synthesis process occurs in fibroblasts via intracellular (messenger RNA transcription, translation, and posttranslational modification) and extracellular (propeptide cleavage and collagen fibril assembly) steps. The primary amino acid sequence of collagen is glycine-proline-X, or glycine-X-hydroxyproline, among which X can be any of the other amino acids, and every third amino acid is glycine (the smallest amino acid). The presence of a glycine at every third position is essential for the formation of the triple helix, which is located at the center of the molecule [3, 5]. 

There are currently 28 distinct types of collagens, of which I to V are the most prevalent. Interestingly, 90% of the collagen in the human body is type I and can be found in the skin, tendons, vessels, bones, and internal organs. Type II collagen is the main component of cartilage, and type III collagen is the main component of reticular fibers and occurs in smooth muscle, the endoneurium and the trabeculae of hematopoietic organs. Type IV collagen comprises the basal lamina, and type V collagen is found in the placenta, hair, and cell surfaces [3, 5]. 

Most dominant negative variants in collagen genes are caused by the substitution of one of the glycines in the collagenous domains of the α chains by a larger amino acid. These glycine substitutions may cause diseases with different phenotypes, from osteogenesis imperfecta (OI) and subtypes of Ehlers–Danlos syndrome (EDS) to chondrodysplasia and Alport syndrome [3]. Table 1 lists specific collagen diseases that can present with musculoskeletal issues and may require rheumatologic and/or orthopedic evaluation.

**Table 1:** Hereditary collagen-related disorders with signs and/or symptoms that may prompt rheumatologic and/or orthopedic consultation

Collagen type	Alpha chain	Disease	OMIM condition ^[12]^	Inheritance	2023 Skeletal dysplasia nosology ^[8]^
Type 1	α1	Caffey disease	114000	AD	NOS 25-0030
		Combined osteogenesis imperfecta and Ehlers-Danlos syndrome 1	619115	AD	
		Ehlers-Danlos syndrome, arthrochalasia type, 1	130060	AD	
		Osteogenesis imperfecta, non-deforming	166200	AD	NOS 26-0010
		Osteogenesis imperfecta, severe perinatal	166210	AD	NOS 26-0030
		Osteogenesis imperfecta, progressively deforming	259420	AD	NOS 26-0080
		Osteogenesis imperfecta, moderate	166220	AD	NOS 26-0270
	α2	Combined osteogenesis imperfecta and Ehlers-Danlos syndrome 2	619120	AD	
		Ehlers-Danlos syndrome, arthrochalasia type, 2	617821	AD	
		Ehlers-Danlos syndrome, cardiac valvular type	225320	AR	
		Osteogenesis imperfecta, non-deforming	166200	AD	NOS 26-0020
		Osteogenesis imperfecta, severe perinatal	166210	AD	NOS 26-0040
		Osteogenesis imperfecta, progressively deforming	259420	AD	NOS 26-0090
		Osteogenesis imperfecta, moderate	166220	AD	NOS 26-0280
Type 2	α1	Achondrogenesis	200610	AD	NOS 02-0010
		Hypochondrogenesis	200610	AD	NOS 02-0020
		Dysplasia of the proximal femoral epiphyses (Avascular necrosis of the femoral head)	608805	AD	NOS 02-0100
		Spondyloepiphyseal dysplasia with metatarsal shortening (Czech dysplasia)	609162	AD	NOS 02-0080
		Kniest dysplasia	156550	AD	NOS 02-0060
		Dysplasia of the proximal femoral epiphyses (Legg-Calve-Perthes disease)	150600	AD	NOS 02-0100
		Spondyloepiphyseal dysplasia congenita (Osteoarthritis with mild chondrodysplasia)	604864	AD	NOS 02-0040
		Platyspondylic dysplasia, Torrance type	151210	AD	NOS 02-0030
		Spondyloepiphyseal dysplasia congenita	183900	AD	NOS 02-0040
		Spondylometaepiphyseal dysplasia, Strudwick type	184250	AD	NOS 02-0050
		Spondyloepiphyseal dysplasia, Stanescu type	616583	AD	NOS 02-0050
		Spondyloperipheral dysplasia	271700	AD	NOS 02-0070
		Stickler syndrome, type I	108300	AD	NOS 02-0090
		Stickler syndrome, type I, nonsyndromic ocular	609508	AD	
Type 3	α1	Ehlers-Danlos syndrome, vascular type	130050	AD	
		Polymicrogyria with or without vascular-type Ehlers-Danlos syndrome	618343	AR	
Type 5	α1	Ehlers-Danlos syndrome, classic type, 1	130000	AD	
		Fibromuscular dysplasia, multifocal	619329	AD	
	α2	Ehlers-Danlos syndrome, classic type, 2	130010	AD	
Type 9	α1	Stickler syndrome, type IV	614134	AR	NOS 09-0080
		Epiphyseal dysplasia, multiple	614135	AD	NOS 09-0050
	α2	Epiphyseal dysplasia, multiple	600204	AD	NOS 09-0060
		Stickler syndrome, type V	614284	AR	NOS 09-0090
	α3	Epiphyseal dysplasia, multiple	600969	AD	NOS 09-0070
		Stickler syndrome, type VI	120270	AR	NOS 09-0100
Type 10	α1	Metaphyseal chondrodysplasia, Schmid type	156500	AD	NOS 11-0010
Type 11	α1	Marshall syndrome	154780	AD	NOS 03-0020
		Stickler syndrome, type II	604841	AD	NOS 03-0010
	α2	Stickler syndrome, non-ocular type	184840	AD	NOS 03-0030
		Otospondylomegaepiphyseal dysplasia, autosomal dominant	184840	AD	NOS 03-0070
		Otospondylomegaepiphyseal dysplasia, autosomal recessive	215150	AR	NOS 03-0060
Type 27	α1	Steel syndrome	615155	AR	NOS 13-0300

AD: autosomal dominant; AR: autosomal recessive

This Review delves into clinical and therapeutic aspects of hereditary connective tissue disorders of most interest for those involved in the care of patients with systemic diseases and incorporates the new nomenclature following the 2023 Nosology of Skeletal Dysplasias publication [8]. The main focus of the manuscript is about establishing a clinical suspicion. Additional investigation, including genetic testing, may be ordered to each disease. It is important for non-specialists to understand that not all patients will have a positive genetic test, and that a negative genetic test does not exclude the possibility of the disease. With this rationale, ordering genetic testing should be reserved to physicians experienced with the different tests and their appropriate interpretation. Genetic counseling is always required in the setting of these rare diseases.

## Osteogenesis imperfecta

Osteogenesis imperfecta is characterized by impaired bone formation and strength, leading to bone fragility. It occurs worldwide and affects all races and ethnicities. Its prevalence ranges from 1 in 10,000 to 1 in 20,000 live births. The disease has a variable course, ranging from mild to severe and lethal. Clinical manifestations include bone fragility, with bone fractures secondary to mild trauma or even spontaneously; short stature; bone deformities, that contribute to impaired walking, thoracic restriction, and dyspnea; brittle teeth; impaired hearing; and joint hyperextensibility [9, 10]. Blue sclerae may be seen in OI but also in Hypermobility Spectrum Disorders/EDS and Marfan syndrome. Additionally, other diseases not comprising collagen and collagen-related genetic diseases may present with blue sclerae, such as iron deficiency or drug toxicity.

Ancillary exams are not specific. Imaging, for example radiography and bone densitometry, is important for fracture diagnosis and follow-up. Genetic testing should be sought for specific diagnosis and counseling, as inheritance patterns may be autosomal dominant, autosomal recessive or X-linked [10]. Table 2 shows the most up-to-date classification [8], in which OI is divided into 4 different phenotypes (non-deforming, moderate, progressively deforming, and severe perinatal forms), consistent with the original phenotypic classification by Sillence et al. [11], and combining the new genetic data with previous expanded Sillence classification (from I to XXIII), with descriptors and Online Mendelian Inheritance in Man (OMIM) numbers [12]. 

**Table 2:** Classification of Osteogenesis Imperfecta According to the 2023 Skeletal Dysplasia Nosology [8]

OI Type	Inheritance	Previous classification	Current classification (2023 Skeletal dysplasia nosology) [8]	Gene	2023 Skeletal dysplasia nosology code [8]	OMIM condition [12]
**Defects in collagen synthesis, structure, or processing**						
I	AD	Mild	Non-deforming	*COL1A1*	NOS26-0010	166200
			Non-deforming	*COL1A2*	NOS26-0020	166200
II	AD	Lethal	Severe perinatal	*COL1A1*	NOS26-0030	166210
			Severe perinatal	*COL1A2*	NOS26-0040	166210
III	AD	Progressive Deforming	Progressively deforming	*COL1A1*	NOS26-0080	259420
			Progressively deforming	*COL1A2*	NOS26-0090	259420
IV	AD	Moderate	Moderate	*COL1A1*	NOS26-0270	166220
			Moderate	*COL1A2*	NOS26-0280	166220
XIII	AR	Mild/Severe	Progressively deforming	*BMP1*	NOS26-0180	614856
**Defects in bone mineralization**						
V	AR	Variable, Distinctive Histology	Progressively deforming	*IFITM5*	NOS26-0100	610967
			Moderate	*IFITM5*	NOS26-0300	166220
			OI With calcification of interosseous membranes and/or hypertrophic callus	*IFITM5*	NOS26-0350	610967
VI	AR	Moderate/Severe	Progressively deforming	*SERPINF1*	NOS26-0110	613982
**Defects in collagen modification**						
VII	AR	Lethal (Null)/ Severe/ Severe (Hypomorphic)	Severe perinatal	*CRTAP*	NOS26-0050	610682
			Progressively deforming	*CRTAP*	NOS26-0120	610682
			Moderate	*CRTAP*	NOS26-0310	610682
VIII	AR	Lethal	Severe perinatal	*P3H1 (LEPRE1)*	NOS26-0060	610915
		Severe	Progressively deforming	*P3H1 (LEPRE1)*	NOS26-0130	610915
IX	AR	Lethal	Severe perinatal	*PPIB*	NOS26-0070	259440
			Progressively deforming	*PPIB*	NOS26-0140	259440
		Moderate	Moderate	*PPIB*	NOS26-0320	259440
XIV	AR	Severe	Progressively deforming	*TMEM38B*	NOS26-0170	615066
**Defects in collagen folding and cross-linking**						
X	AR	Severe/Lethal	Progressively deforming	*SERPINH1*	NOS26-0150	613848
XI / BRKS1	AR	Severe	Progressively deforming	*FKBP10*	NOS26-0160	610968
		Mild	Moderate	*FKBP10*	NOS26-0330	610968
		Bruck syndrome type 1	Bruck syndrome type 1	*FKBP10*	NOS26-0430	259450
BRKS2	AR	Bruck syndrome type 2	Bruck syndrome type 2	*PLOD2*	NOS26-0440	609220
**Defects in osteoblast development with collagen insufficiency**						
XII	AR	Severe	Moderate	*SP7*	NOS26-0340	613849
XV	AR	Severe	Progressively deforming	*WNT1*	NOS26-0190	615220
			Moderate	*WNT1*	NOS26-0290	166220
XVI	AR	Severe	Progressively deforming	*CREB3L1*	NOS26-0200	616229
XVII	AR	Progressive Severe	Progressively deforming	*SPARC*	NOS26-0210	616507
XVIII	XLR	Moderate/Severe	Progressively deforming	*MBTPS2*	NOS26-0230	301014
XIX	AR	Severe	Progressively deforming	*FAM46A/* *TENT5A*	NOS26-0220	617952
XX	AR	Progressive Severe/Lethal	Progressively deforming	*MESD*	NOS26-0240	618644
XXI	AR	Severe + Neurodevelopmental	Progressively deforming with neurodevelopmental features	*KDELR2*	NOS26-0250	619131
XXII	AR	Severe	Progressively deforming	*CCDC134*	NOS26-0260	619795
**Other bone fragility disorders**						
	AD		OI with craniosynostosis (Cole Carpenter syndrome)	*P4HB*	NOS26-0360	112240
	AR		OI with craniosynostosis (Cole Carpenter syndrome)	*SEC24D*	NOS26-0370	616294
	XL		Osteoporosis X-linked form	*PLS3*	NOS26-0380	300910
	XL		Osteoporosis X-linked form	*MBTPS2*	NOS26-0390	301014
	AD		Osteoporosis - dominant form	*WNT1*	NOS26-0400	615220
	AD		Osteoporosis - dominant form	*LRP5*	NOS26-0410	166710, 601884
	AD		Osteoporosis - dominant form	*ARHGAP25*	NOS26-0420	610587
	AR		Osteoporosis-pseudoglioma syndrome	*LRP5*	NOS26-0450	259770
	AD		Bone fragility with calvarial “doughnut” lesions	*SGMS2*	NOS26-0460	126550

AR: autosomal recessive; AD: autosomal dominant; XL: X-linked

Patients with OI require interdisciplinary and personalized treatment comprising both clinical and surgical approaches. Supportive clinical treatment includes dental care, audiological care, rehabilitation, physical therapy, pain modulation, and bone-targeted drugs, such as bisphosphonates, to increase bone mineral density and reduce fracture risk, although not conclusively. There is currently no compelling evidence to support the use of teriparatide in the treatment of osteogenesis imperfecta. Improvement in bone mineral density is apparent, but this observation was limited to patients with OI type I. Protocols with romosozumab in humans with OI are lacking but a different sclerostin inhibitor is currently being studied in these patients – sestrusumab. Devices to assist mobility and prevent falls may be needed. Surgical procedures are directed at improvement of patient growth and development, correction and/or prevention of deformities and enhancement of quality of life. Surgical interventions include intramedullary rod placement, surgery to manage basilar invagination, and correction of scoliosis [9, 13, 14]. 

The prognosis for OI varies in accordance with the severity of the disease and the response to treatment. Patients may lead active, productive lives with appropriate treatment. However, in severe cases, bone fragility can significantly impose limitations on daily living activities and cause complications [9]. Importantly, physicians caring for these patients must include as differential other genetic diseases, such as hypophosphatasia, and child maltreatment.

## Hypermobility spectrum disorder/Ehlers‒Danlos syndrome

Hypermobility spectrum disorders (HSD) are connective tissue disorders that cause joint hypermobility, instability, injury, and recurrent, persistent, and/or chronic pain. HSD is diagnosed by medical history and physical examination, and this terminology should not be used for asymptomatic persons. Physicians should inquire about their history of joint luxation and/or subluxation, early cartilage lesions or evidence of early-onset osteoarthritis and recurrent soft tissue lesions (sprains, tendinopathies) in addition to chronic pain. Physical examination should encompass assessment of proprioception and the use of the Beighton [15] criteria, assuming age and sex influences, to help establish a clinical diagnosis. Notably, hypermobile joints may not always have a specific relationship with collagen disorders since they can be identified in distinct genetic disorders, for example: Down syndrome (trisomy 21), homocystinuria (cystathionine β-synthase deficiency) and mucopolyssacaridosis type VI (lysosomal hydrolase N-acetylgalactosamine 4-sulfatase deficiency) [16–18]. 

Some patients may definitely have Ehlers–Danlos syndrome, corroborating the updated language that considers HSD and EDS a continuum [19]. EDS is a rare condition with different prevalence according to subtypes that comprise 13 different phenotypes, each with distinct diagnostic criteria. It is estimated to affect 1 in 5,000 people worldwide. The most common subtype is hypermobile EDS, which lacks association with a specific gene. In addition to musculoskeletal signs and symptoms, the clinical manifestations of EDS can vary significantly between subtypes and include skin hyperextensibility, easy bruising, and abnormal scarring; moreover, some patients may experience viscus rupture, spontaneous arterial dissection, or aneurysm formation. The clinical circular (not linear) continuum also includes aspects such as fatigue, headache, anxiety, gastrointestinal problems, mast cell activation disorder, and autonomic dysfunction [17, 18, 20]. 

Like OI, HSD/EDS is diagnosed on clinical grounds, and supplementary exams are not specific. Imaging studies will evaluate musculoskeletal manifestations such as osteoarthritis, scoliosis, cervical instability, normal or decreased bone density, and heart and vascular disorders, that is heart valve disease, rhythm disturbances, autonomic disorders, arterial enlargement, and dissection [9, 20]. Genetic studies may identify a background that enables counseling. Table 3 depicts the 2017 nomenclature and genes associated with EDS [17]. Interestingly, spondylodysplastic and musculocontractural EDS are also considered at the 2023 Skeletal Dysplasia Nosology [8]; and periodontal EDS, is contemplated at the 2022 Update on the Classification from the International Union of Immunological Societies Expert Committee [21]. 

**Table 3:** Classification of Ehlers–Danlos Syndrome [16]

Clinical EDS subtype	Inheritance	Group	Protein	Gene	2023 Skeletal dysplasia nosology code [8]	OMIM Condition [12]
1 Classical EDS (cEDS)	AD	Defects in collagen primary structure and collagen processing	Type V collagen	*COL5A1*		
			Type V collagen	*COL5A2*		130010
			Type I collagen	Rare: *COL1A1* c.934C>T, p.(Arg312Cys)		
2 Classical-like EDS (clEDS)	AR	Defects in structure and function of myomatrix, the interface between muscle and ECM	Tenascin XB	*TNXB*		606408
3 Cardiac-valvular EDS (cvEDS)	AR	Defects in collagen primary structure and collagen processing	Type I collagen	*COL1A2* (biallelic variants that lead to *COL1A2* NMD and absence of pro α2(I) collagen chains)		225320
4 Vascular EDS (vEDS)	AD	Defects in collagen primary structure and collagen processing	Type III collagen	*COL3A1*		130050
			Type I collagen	*COL1A1* c.934C>T, p.(Arg312Cys) c.1720C>T, p.(Arg574Cys) c.3227C>T, p.(Arg1093Cys)		
5 Hypermobile EDS (hEDS)	AD	Unknown	Unknown	Unknown		130020
6 Arthrochalasia EDS (aEDS)	AD	Defects in collagen primary structure and collagen processing	Type I collagen	*COL1A1*		130060
				*COL1A2*		130060
7 Dermatosparaxis EDS (dEDS)	AR	Defects in collagen primary structure and collagen processing	ADAMTS-2	*ADAMTS2*		225410
8 Kyphoscoliotic EDS (kEDS)	AR	Defects in collagen folding and collagen cross-linking	Lysylhydroxylase 1	*PLOD1*		225400
			FKBP22	*FKBP14*		614557
9 Brittle Cornea syndrome (BCS)	AR	Disorders of intracellular processes	ZNF469	*ZNF469*		229200
			PRDM5	*PRDM5*		614170
10 Spondylodysplastic EDS (spEDS)	AR	Defects in glycosaminoglycan biosynthesis	β4GalT7	*B4GALT7*	NOS05-0070	130070, 615349
			β3GalT6	*B3GALT6*	NOS05-0060*	271640
		Disorders of intracellular processes	ZIP13	*SLC39A13*	NOS13-0230	612350
11 Musculocontractural EDS (mcEDS)	AR	Defects in glycosaminoglycan biosynthesis	D4ST1	*CHST14*	NOS04-0090	601776
			DSE	*DSE*	NOS04-0100	615539
12 Myopathic EDS (mEDS)	AD or AR	Defects in structure and function of myomatrix, the interface between muscle and ECM	Type XII collagen	*COL12A1*		616471
13 Periodontal EDS^#^ (pEDS)	AD GOF	Defects in complement pathway	C1r	*C1R*		130080
			C1s	*C1S*		130080

AD: autosomal dominant; AR: autosomal recessive; GOF: gain of function

* Also termed Spondyloepimetaphyseal dysplasia with joint laxity (Beighton type), *B3GALT6-*related.

^#^ Also considered as Inborn Errors of Immunity (complement deficiencies) [20]

HSD/EDS treatment is aimed at symptom relief, damage prevention, and quality of life improvement. These interventions may include rehabilitation, physical and occupational therapies, analgesics, pain modulators and short courses of nonsteroidal anti-inflammatory drugs to relieve pain. Beta blockers are recommended for patients with vascular EDS to prevent arterial enlargement and may be indicated for dysautonomia as an option for postural orthostatic tachycardia syndrome. Surgery is reserved for severe dislocations and vascular or internal organ complications. Psychological care plays a significant role in these patients [18, 20, 22]. 

The prognosis varies with the severity of the disease and the impact of the manifestations on daily and professional activities [20]. 

## Marfan syndrome

Marfan syndrome (NOS 31-0010) is a type 1 fibrillinopathy caused by pathogenic variants in *FBN1* gene, which encodes fibrillin-1. Not all type 1 fibrillinopathies manifest marfanoid features. The fibrillin-1 protein is important for the extracellular matrix underlying arteries, pericondrium and eye structures. The disease can occur *de novo* in 25% of the patients or be inherited in an autosomal dominant pattern. The prevalence of the carrying status of *FBN1* is estimated to be 6.5 cases per 100,000 persons [23–26]. 

Clinical signs and symptoms include aortic root aneurysm, aortic dissection, ectopia lentis, and skeletal abnormalities/marfanoid habitus, inclusive of high stature, disproportionally long limbs (arm span-to-height ratio > 1.05), pectus excavatum or carinatum, arachnodactyly, and scoliosis [27]. The revised Ghent diagnostic criteria aid in the identification of these patients. Table 4 shows the MFS diagnostic criteria [26]. 

**Table 4:** Revised Ghent Diagnostic Criteria for Marfan Syndrome [26]

Systemic Features Scoring (maximum: 20 points; score ≥ 7 indicates systemic involvement)								
Wrist AND thumb signs^#^								3
Wrist OR thumb sign^#^								1
Pectus carinatum deformity								2
Pectus excavatum or chest asymmetry								1
Hindfoot deformity								2
Plain pes planus								1
Pneumothorax								2
Dural ectasia								2
Protrusio acetabuli								2
Reduced upper segment/lower segment ratio AND increased arm/height AND no severe scoliosis								1
Scoliosis or thoracolumbar kyphosis								1
Reduced elbow extension								1
Facial features (3/5) dolichocephaly, enophthalmos, downslanting palpebral fissures, malar hypoplasia, retrognathia								1
Skin striae								1
Myopia > 3 diopters								1
Mitral valve prolapse (all types)								1
Diagnostic criteria*								
**Family History Negative**								
**Marfan Syndrome**	Aortic diameter at the sinuses of Valsalva ≥ Z-score 2, or aortic root dissection	+	Ectopia Lentis					
			*FBN1*					
			Systemic Features ≥ 7 points					
	Ectopia Lentis	+	*FBN1* variant **with** known association with aortic enlargement or dissection					
**Ectopia Lentis Syndrome**	Ectopia Lentis	+	*FBN1* variant **without** known association with aortic enlargement or dissection	+	Systemic Features			
		–		–				
**MASS**	Aortic diameter at the sinuses of Valsalva < Z-score 2	+	Systemic Features ≥ 5 points (at least one skeletal feature)	–	Ectopia Lentis			
**MVPS**	Mitral Valve Prolapse	+	Aortic diameter at the sinuses of Valsalva < Z-score 2	+	Systemic Features < 5 points	–	Ectopia Lentis	
**Family History Positive**								
**Marfan Syndrome**	Positive Family History	+	Ectopia Lentis					
			Systemic Features ≥ 7 points					
			Aortic diameter at the sinuses of Valsalva ≥ Z-score 2, above age 20 years					
			Aortic diameter at the sinuses of Valsalva ≥ Z-score 3, below age 20 years					

MASS: myopia, mitral valve prolapse, borderline (Z-score < 2) aortic root dilatation, striae, skeletal findings phenotype

MVPS: mitral valve prolapse syndrome

# The “wrist sign” is positive when the thumb overlaps the fifth finger when grasping the contralateral wrist. The “thumb sign” is positive when it protrudes beyond the edge of the clenched fist

* Individuals with features suggestive of other syndromes, such as Loeys-Dietz syndrome, Shprintzen-Goldberg syndrome, congenital contractural arachnodactyly, familial thoracic aortic aneurysms, and dissection, and vascular Ehlers-Danlos syndrome, need to have these diagnoses excluded through genetic testing

Adequate diagnosis and prophylactic usage of β-blockers can prevent aneurysm enlargement and aortic dissection. Angiotensin-II receptor blockers, either alone or in combination with β-blockers, also play a role in the prevention of aortic growth in patients with MFS. Regular imaging and lifestyle modifications are the first steps for the protection of the aorta. Ophthalmological care is warranted. Prevention of additional cardiovascular risk factors should be encouraged. Elective surgery is indicated in the case of aortic root dilatation of ≥ 5 mm/year or an absolute aortic diameter of ≥ 50 mm at any level. Surgical stabilization of the spinal deformity should be considered when the curve progresses beyond 40 degrees. Retinal detachment needs to be diagnosed early and can be managed with laser surgery, vitrectomy, or scleral buckle surgery according to the surgeon’s indications. If the lens is dislocated to the extent that the patient’s vision cannot be corrected through the lens, the risk, benefit, and timing of removal of the dislocated lens must be carefully considered. Bone density should be assessed but it is infrequently a significant issue [22, 24, 27, 28]. 

## Loeys–Dietz syndrome

Loeys–Dietz syndrome (LDS) is a TGFβ signaling pathway condition that affects multiple organ systems and comprises different subtypes, all of which exhibit autosomal dominant inheritance (Table 5). This syndrome is an important differential syndrome for patients with a Marfan-like phenotype not fulfilling the MFS diagnostic criteria, usually with aortic and skeletal features but without ectopia lentis, and for patients reminiscent of vascular EDS, with negative *COL3A1* genetic testing. The risk for arterial dissection in these patients may be greater than that in patients with MFS, and arterial dissection may occur at a younger age [22, 29–31]. 

**Table 5:** Classification of Loeys–Dietz syndrome

LDS type	Gene	OMIM [12]	Chromosome	2023 Skeletal dysplasia nosology [8]
LDS1	*TGFBR1*	609192	9q22	NOS 31-0030
LDS2	*TGFBR2*	610168	3p24	NOS 31-0040
LDS3	*SMAD3*	613795	15q	NOS 31-0080
LDS4	*TGFB2*	614816	1q41	NOS 31-0050
LDS5	*TGFB3*	615582	14q24	NOS 31-0060
LDS6	*SMAD2*	619656	18q21	NOS 31-0070

LDS1 (*TGFBR1*-related) is characterized by arterial tortuosity and/or aneurysms, most frequently of the aorta, combined with craniofacial abnormalities, including hypertelorism, bifid uvula or cleft palate. Arachnodactyly, pectus deformity, retrognathia, and joint laxity may be additional clinical features. LDS2 (*TGFBR2*‐related) is characterized by arterial tortuosity, aortic aneurysms, hypertelorism, abnormal uvula, joint laxity, pectus deformity, scoliosis, arachnodactyly, and malar hypoplasia. Notably, LDS2 is characterized by minimal craniofacial abnormalities compared to LDS1 [29, 30]. 

LDS3 (*SMAD3*-related) is characterized by early-onset osteoarthritis and osteochondritis dissecans. Additionally, patients may exhibit scoliosis, aortic aneurysms, cerebral arterial tortuosity, mitral valve prolapse, and abnormal uvula and palate. Most patients develop osteoarthritis by the fourth decade of life, with frequent involvement of the hands, wrists, knees, and cervical and lumbar spine, with intervertebral disc degeneration [29, 30]. 

LDS4 (*TGFB2*-related) patients may present with aortic aneurysms and dissection, joint laxity, pectus deformities, scoliosis, arachnodactyly, pes planus, high-arched palate, or umbilical/inguinal hernias. The few patients diagnosed with LDS5 (*TGFB3*‐related) were described as having aortic aneurysms with a risk of dissection, arachnodactyly, pectus deformity, pes planus or clubfeet, hypertelorism, abnormal uvula, joint laxity, cervical spine instability and skeletal overgrowth [29, 30]. 

LDS6 (*SMAD2*-related) was described more recently in 4 patients from 3 unrelated families. Clinical signs and symptoms included aortic, carotid, and cerebral artery aneurysms; dysmorphic features; and skeletal and skin abnormalities [30]. 

Early diagnosis warrants medical therapy and vascular surgery procedures to prevent arterial enlargement and dissection and orthopedic care to correct skeletal deformities [22]. Physical and occupational therapies aid in the supportive management of these patients. Low bone density with a propensity for fractures may be observed in all LDS variants, and specific therapy may be necessary [28]. 

### Stickler syndrome

Stickler syndrome (STL) is a condition with a heterogeneous clinical presentation and genetic background characterized by ocular, auditory, skeletal, and orofacial manifestations [32]. Patients exhibit both autosomal dominant (STL1, *COL11A1*-related; and STL2, *COL11A2*-related; and *COL11A2*-related non-ocular type) [33] and autosomal recessive (STL4, *COL9A1*-related; and STL5, *COL9A2*-related; and STL6, *COL9A3*-related) [34] inheritance (Table 1). STL3 was reclassified as otospondylomegaepiphyseal dysplasia, *COL11A2*-related, and both types of autosomal inheritance were detected [8]. 

Patients with most forms of STL exhibit ocular changes, including high myopia, vitreoretinal degeneration, and cataracts. Notably, it is the most common cause of retinal detachment in children and the most common cause of familial or inherited retinal detachment. These patients may also present cleft palate, bifid uvula, Pierre Robin sequence (micrognathia, glossoptosis, and airway obstruction), flat midface, and hearing impairment. Multiple epiphyseal dysplasias and early-onset osteoarthritis are common musculoskeletal features [32, 35]. 

Treatment includes assessment by a craniofacial specialist and/or otolaryngologist, an ophthalmologist, a feeding specialist, and physical and occupational therapies. Preventative surgery is effective at prevention of retinal detachment in patients at substantial risk [32, 36]. 

## Conclusion

Hereditary connective tissue disorders may be rare but can be recognized whenever physicians, particularly rheumatologists, consider the combination of signs and symptoms that, despite arising from different organs and tissues, can have a parallel genetic background. A multidisciplinary and multispecialty approach is fundamental for the adequate care of these patients. Knowledge of basic genetic concepts is important for nonspecialists to aid in the curtailment of the patients’ journeys and the reduction of misdiagnosis.

## Data Availability

Data sharing not applicable – no new data generated.
